# Interchangeability of biosimilars: A study of expert views and visions regarding the science and substitution

**DOI:** 10.1371/journal.pone.0262537

**Published:** 2022-01-11

**Authors:** Louise C. Druedahl, Sofia Kälvemark Sporrong, Timo Minssen, Hans Hoogland, Marie Louise De Bruin, Marco van de Weert, Anna Birna Almarsdóttir

**Affiliations:** 1 Copenhagen Centre for Regulatory Science (CORS), Department of Pharmacy, Faculty of Health and Medical Sciences, University of Copenhagen, Copenhagen, Denmark; 2 Social and Clinical Pharmacy, Department of Pharmacy, Faculty of Health and Medical Sciences, University of Copenhagen, Copenhagen, Denmark; 3 Centre for Advanced Studies in Biomedical Innovation Law (CeBIL), Faculty of Law, University of Copenhagen, Copenhagen, Denmark; 4 Social Pharmacy, Department of Pharmacy, Faculty of Pharmacy, Uppsala University, Uppsala, Sweden; 5 LEO Pharma A/S, Ballerup, Denmark; 6 Utrecht Institute for Pharmaceutical Sciences, Utrecht University, Utrecht, The Netherlands; 7 Drug Delivery and Biophysics of Biopharmaceuticals, Department of Pharmacy, Faculty of Health and Medical Sciences, University of Copenhagen, Copenhagen, Denmark; Shanghai Jiao Tong University Medical School Affiliated Ruijin Hospital, CHINA

## Abstract

Healthcare systems have reached a critical point regarding the question of whether biosimilar substitution should become common practice. To move the discussion forward, the study objective was to investigate the views of experts from medicines agencies and the pharmaceutical industry on the science underpinning interchangeability of biosimilars. We conducted an empirical qualitative study using semi-structured interviews informed by a cross-disciplinary approach encompassing regulatory science, law, and pharmaceutical policy. In total 25 individuals with experience within biologics participated during September 2018–August 2019. Eight participants were EU national medicines authority regulators, and 17 had pharmaceutical industry background: five from two originator-only companies, four from two companies with both biosimilar and originator products, and eight from seven biosimilar-only companies. Two analysts independently conducted inductive content analysis, resulting in data-driven themes capturing the meaning of the data. The participants reported that interchangeability was more than a scientific question of likeness between biosimilar and reference products: it also pertained to regulatory practices and trust. Participants were overall confident in the science behind exchanging biosimilar products for the reference products via switching, i.e., with physician involvement. However, their opinions differed regarding the scientific risk associated with biosimilar substitution, i.e., without physician involvement. Almost all participants saw no need for additional scientific data to support substitution. Moreover, the participants did not believe that switching studies, as required in the US, were appropriate for obtaining scientific certainty due to their small size. It is unclear why biosimilar switching is viewed as scientifically safer than substitution; therefore, we expect greater policy debate on biosimilar substitution in the near future. We urge European and UK policymakers and regulators to clarify their visions for biosimilar substitution; the positions of these two frontrunners are likely to influence other jurisdictions on the future of biosimilar use.

## Introduction

Biologics are important for clinical practice because they offer targeted treatments within complex disease areas, such as rheumatology and dermatology, and are considered key for the present and next generation of medicines [1, 2]. However, the products are costly and enhancing competition in the biologics sector has potential to provide substantial savings [3–5]. Biosimilars are follow-on products whose quality, safety and efficacy are highly similar compared and without clinical meaningful differences to that of the biological reference product. Thus, biosimilars are highly similar versions of the active substance of the originator reference product [6]. Nevertheless, debates are ongoing on the safe exchange between reference products and biosimilars in clinical practice [7–14].

In clinical settings in the EU, the decision to exchange (termed *interchange*) a reference product for a biosimilar can be taken by either a physician (termed *switching*) or a pharmacist (termed *substitution*) [15]. In the UK definition, *interchangeable* in the context of biosimilars means an exchange conducted via switching [16], see Table 1. While switching of biologics is relatively common in the EU, substitution is not common practice [17]. This stands in contrast to small molecule-generics, which via substitution have led to considerable cost reduction in European healthcare systems [18, 19]. The difference in clinical practice between generics and biosimilars is ascribed to the perceived risks related to biologics being larger, more complex molecules that are usually impossible to produce in exact copies [5, 20]. However, in a systematic review of 90 studies investigating switching between biosimilar and reference products, only one study showed loss of efficacy (but no adverse effects) for one in 17 patients, and one study showed increased patient discontinuation rates after switching from reference to biosimilar product [21]. The effects of multiple switching between biologic products remain largely uninvestigated [21, 22].

**Table 1:** Definitions, requirements and regulatory level of responsibility of biosimilarity, interchangeability, switching and substitution in the EU, the UK and the US.

	The EU [6, 15, 23]	The UK [24]	The US [25, 26]
**Biosimilarity**	Definition:	Definition:	Definition:
	A biosimilar needs to have biosimilarity to the reference product, i.e. be highly similar in terms of efficacy, safety and quality.	In principle the same as for the EU	In principle the same as for the EU
	
	Requirements:		
		Requirements:	Requirements:
	Biosimilar marketing authorization, i.e. legal and regulatory requirements	Biosimilar marketing authorization, i.e. legal and regulatory requirements	Biosimilar marketing authorization, i.e. legal and regulatory requirements
	Regulatory level:	Regulatory level:	Regulatory level:
	European Medicines Agency and European Commission	Medicines Healthcare products Regulatory Agency, the UK	US Food and Drug Administration
**Interchangeability**	Definition:	Definition:	Definition:
	“exchanging one medicine for another medicine that is expected to have the same clinical effect” [15]	“Once a biosimilar is authorised, it is considered interchangeable with the RP, which means that a prescriber can choose the biosimilar over the RP (or vice versa) and expect to achieve the same therapeutic effect.” [24]	“the term interchangeable or interchangeability, in reference to a biological product/…/means that “the biological product may be substituted for the reference product without the intervention of the health care provider who prescribed the reference product.” [25]
	Requirements:	Requirements:	Requirements:
	No central EU requirements	No regulatory requirements	Interchangeable designation obtained via regulatory requirements
	Regulatory level:		Regulatory level:
	EU member states		US Food and Drug Administration
**Switching**	Definition:	Definition:	Definition:
	“when the prescriber	In principle the same as for the EU.	In principle the same as for the EU.
	decides to exchange one medicine for another medicine with the same therapeutic intent” [15]		
	Requirements:	Requirements:	Requirements:
	No central EU requirements	No regulatory requirements	No regulatory requirements
		National guidance	
	Regulatory level:		Regulatory level:
	EU member states		State level
**Substitution**	Definition:	Definition:	Definition:
	“dispensing one medicine instead of another equivalent and interchangeable medicine at pharmacy level without consulting the prescriber” [15]	In principle the same as for the EU.	In principle the same as for the EU.
	Requirements:	Requirements:	Requirements:
	No central EU requirements	“Substitution at the pharmacy level without consulting the prescriber is not permitted for biological medicines, including biosimilars” [24]	Product-level interchangeability designation by the US Food and Drug Administration
	Regulatory level:		
			Regulatory level:
	EU member states		US Food and Drug Administration
			State level laws

Healthcare systems have reached a critical point regarding the question of whether biosimilar substitution should become common practice. There appears no clear way forward as contrasting views exist on the scientific appropriateness of biosimilar substitution among both the industry and European national medicines agencies, and there are varying practices within the EU regarding the roll out of biosimilars in the member states’ health care systems [17, 27, 28]. Similarly, the UK does not currently allow substitution of biologics following a biosimilar approval by its Medicines & Healthcare products Regulatory Agency (MHRA) [24]. In the EU, the European Medicines Agency (EMA) is the authority assessing the marketing authorization for most biologics under the centralized procedure in the current European regulatory framework [29, 30], and biosimilar marketing applications are centrally discussed at the EMA following assessments carried out by experts affiliated to EU national medicines agencies. However, all decisions regarding interchangeability (thus switching and substitution) of approved medicines fall strictly under the member states’ national sovereignty [15]. A different approach to interchangeability is taken by the US Food and Drug Administration (FDA) where a company can apply for an interchangeability designation for their biosimilar product at the same time as or after the marketing authorization [25, 31]. To obtain interchangeability status, the US FDA requires clinical switching studies to prove that multiple switching of the reference and biosimilar product does not result in increased risks for patients [25, 31]. The presence of widely different approaches in important jurisdictions seems to complicate how much evidence is needed for biosimilars to be substituted in the clinic. To move the discussion on interchangeability forward, we investigated the views of experts from medicines agencies and the pharmaceutical industry on the science underpinning interchangeability of biosimilars. From this it is unclear why biosimilar switching is viewed as scientifically safer than substitution; therefore, we expect greater policy debate on biosimilar substitution in the near future.

## Methods

### Research design

The aim of the study requires data collection of expert knowledge. Therefore, we adopted a qualitative approach as it is appropriate for accessing such knowledge [32]. Empirical data were collected through semi-structured, in-depth interviews [33]. The study was conducted within regulatory science and was informed by the cross-disciplinary expertise among the authors. The authors are from the fields of pharmaceutical policy (ABA, LCD, SKS), regulatory science (LCD, MLDB), law (TM), social science (SKS), protein formulation (MvdW) and regulatory affairs within the pharmaceutical industry (HH). LCD, ABA and SKS have prior experience with qualitative research. All authors each hold a PhD or an LLD degree; when the study was conducted, LCD was a PhD candidate. The study is reported in accordance with the reporting guideline for qualitative research COREQ (consolidated criteria for reporting qualitative research) [34].

### Sampling and recruitment

The sampling strategy was purposive and aimed to cover a wide range of information-rich perspectives and experiences. Eligible interviewees included former or current EU or US medicines agency regulators with experience of biologics, and former or current employees of (or consultants to) pharmaceutical companies with EMA/FDA-approved originator biologic product(s), biosimilar product(s), or both types of product. At the time of the study, the UK was still a member of the EU and therefore it was not specifically targeted to recruit UK medicines agency regulators. Interviewees with company experience were purposefully selected to have expertise within chemistry, manufacturing, and control (CMC) processes, and legal or regulatory affairs. The primary recruitment method was networking, but also included snowballing, i.e., the interviewer asked interviewees to suggest other relevant experts to include in the study. The Faculty of Health and Medical Sciences at the University of Copenhagen approved the study (SUND-2018-09). We approached 29 eligible interviewees either in-person or by email. Study information was distributed to all participants; the information included that the study was part of LCD’s doctoral work. Interviewees were not offered any token of incentive and all interviewees provided written informed consent.

### Data collection instrument

An interview guide (S1 Table) was developed based on informal meetings with regulators and industry representatives. The interview guide was semi-structured and used open-ended questions to capture aspects important to the interviewees that were not considered by the authors. The interview guide was designed to investigate the performance of the current regulation of recombinant protein biosimilars; the focus of the present study is on one subset of the data. An article on manufacturing challenges has already been disseminated [35]. The interview guide was pilot tested, which did not result in any changes to the guide.

### Data collection

LCD is female and experienced with qualitative research, and she conducted interviews either face-to-face or by audio call during September 2018–August 2019. The interview location was decided by the interviewee. Interviewees were asked about their personal perspectives based on their current or previous professional experiences and were not interviewed as formal representatives of their workplace. All interviews but one were recorded and transcribed verbatim (extensive notes were taken of the interview that was not recorded). Field notes were made for all interviews to capture the interviewer’s reflexivity on the interview content. LCD validated the transcripts by reading each transcript while listening to the recording. Each interviewee could provide comments and approved the respective transcript or notes.

### Analysis

For the methodological framework, conventional content analysis was used on the textual data in transcripts and notes from the interviews, which is suitable for letting the data rather than researchers’ pre-conceived categories drive the analysis [36]. Two analysts (LCD and SKS) coded the data inductively and independently; LCD coded all the transcripts in NVivo (version 12.6, QSR International) and SKS coded a subset of 18 transcripts on hard copy. LCD and SKS compared their coding to ensure the underlying meaning of all text segments relevant to the aim was captured in codes. On this basis, a data driven list of codes was developed and condensed into themes capturing the meaning of the data. The data driven codes and themes along with several transcripts were audited [36] by ABA. Thereafter, LCD, ABA and SKS discussed and finalized the analysis. All authors contributed to the final, aggregated analysis, enabling nuanced reflections and perspectives.

## Results

Twenty-five individuals from either EU national medicines agencies or the pharmaceutical industry participated in 23 interviews; all interviews were individual, except two, which were conducted with two interviewees each at their request, see interviewee characteristics in Table 2. The median interview time was one hour and two minutes. Supportive quotes, grouped by theme, are shown in Table 3. The participants reflected on interchangeability in a European context and the science needed to support the procurement and use of biosimilars conducted at member state level.

**Table 2:** Interviewee characteristics and those invited that did not participate. Values are number of interviewees.

Interviewee characteristic	Interviewees (n = 25)	Non-participation (n = 4)
**Workplace**		
European national medicines authority^a^	8	1
European medicines authority	0	1
US medicines authority	0	2
Originator-only manufacturers^b^	5	-
Originator and biosimilar manufacturers^c^	4	-
Biosimilar-only manufacturers^d^	8	-
**Primary expertise of company interviewees**		
Regulatory policy/affairs	10	-
Chemistry, manufacturing, and control	3	-
Law	4	-
**Recruitment strategy**		
Networking	18	2
Snowballing	7	2

^a^ From seven different EU countries.

^b^ From two companies.

^c^ From two companies.

^d^ From seven companies.

**Table 3:** Selected quotes from interviewees on interchangeability of biosimilars.

Theme	Illustrative quote
**The science underpinning biosimilar substitution**	*“They [the FDA] opened [up] for a different grade of being similar*. *So they have [that] you can be a biosimilar*, *which indicates you are similar*, *and then you can have an interchangeable biosimilar*, *which indicates you are ‘highly similar’*, *but what parameters is it that need to be so highly similar*? *And they [the FDA] are really struggling to figure this out” (EU national medicines authority regulator) (Quote 1)*
	*“but what if you’d switch every month or every week or twice a week*. *We don’t really know” (EU national medicines authority regulator) (Quote 2)*
	*“Scientifically they’re [switching studies] not particularly informative/…/the sample sizes are so small they can only really pick up big differences/…/It’s hard to imagine that that could have slipped through the analytical characterizations and the functional assay*. *It’s really hard to imagine” (biosimilar company interviewee)*. *(Quote 3)*
	*“even if you collect a clinical study as in the US there will still be a certain level of uncertainty/…/following up in the post-marketing setting is still critical” (originator company interviewee)*. *(Quote 4)*
**Can substitution be expected in Europe?**	*“Whereas EMA has never anticipated that*, *they think it will always be a case of switching*, *the doctors switching over the patients*, *but having oversight*. *So from an EMA perspective they don’t think it’s needed” (originator and biosimilar company participant) (Quote 5)*

### The science underpinning biosimilar switching

For the science underpinning switching, the participating regulators expressed trust in EMA approved and FDA approved biosimilars as being switchable based solely on their approval in one of these jurisdictions. Switching was not considered equally feasible in all cases as some interviewees expressed that initiating biosimilar treatment in treatment-naïve patients was preferable to switching patients already on treatment. This was perceived as a means to avoid uncertainty regarding unwanted effects from a switch. Several interviewees argued for the scientific soundness of biosimilar switching and none mentioned arguments against it.

### The science underpinning biosimilar substitution

For the science underpinning substitution, the participants disagreed with the US approach where two degrees of biosimilarity are established by distinguishing between those that are approved as US interchangeable (i.e., substitutable) and those that are approved as a biosimilar but not US interchangeable with the originator product. Moreover, they questioned how this suggested difference should be demonstrated in practice (Quote 1). In addition, participants saw these two degrees of similarity as unaligned with the European understanding of biosimilarity. Several interviewees pointed out that the issue was understanding the currently unknown, potential risks of biologic substitution. Thus, uncertainty exists about the potential consequences of biosimilar substitution (i.e., multiple switching) (Quote 2).

The participants did not clearly express what scientific evidence was needed to obtain scientific certainty to substitute biosimilars. However, all but one interviewee said that switching studies, as required in the US, are inadequate to demonstrate that biologic substitution does not result in an increased risk. This was argued by the interviewees because they questioned what additional information a switching study could provide that is not known from the regulatory approval process for biosimilars. Some regulators explained that the rationale of switching studies was to investigate potential differences in anti-drug antibodies, but differences are difficult to detect unless the clinical trials are large, and switching studies were perceived as too small for this purpose (Quote 3). An originator company interviewee stressed that there would be a need for large, long-term studies with multiple switches, and that even if studies similar to those done for the US FDA were carried out, uncertainty would remain. According to this interviewee, the solution seemed to be extensive post-marketing follow-up (Quote 4).

### Can substitution be expected in Europe?

None of the company interviewees argued for substitution in pharmacies. A biosimilar company interviewee explained that biosimilar companies were generally against substitution because it could lead to a race-to-the-bottom for prices and that it would not align with companies’ expectations for return on their investment. Regulators varied in their opinions about substitution of biologics. Some regulators questioned whether the current knowledge on multiple switches was sufficient to allow substitution; others argued that there were enough scientific data to support it. One regulator emphasized that pharmacist-led substitution was the long-term goal for biosimilars from a societal perspective. However, according to a participant from an originator and biosimilar company, the EMA did not anticipate biosimilar substitution; instead, it would always be a case of switching (Quote 5).

## Discussion

The results show that our interviewees view interchangeability as more than a scientific question of likeness between biosimilar and reference products: it also pertains to regulatory practices and trust. Participants were overall confident about biosimilar switching. However, their opinions differed about the scientific risk associated with substitution of biosimilars. For science to underpin substitution, almost all saw no need for additional data and one said large post-marketing trials would be needed. Participants did not believe that switching studies were appropriate for obtaining such scientific certainty due to their small size.

Overall, it is unclear why the interviewed experts within EU national medicines agencies and the pharmaceutical industry believe that biosimilar switching is scientifically safer than substitution when they do not believe in the US approach based on two degrees of biosimilarity. A white paper from Australia [37] suggests that the rhetoric around interchangeability is changing from originally being a question of whether repeated switching raises issues, through asking what additional evidence sponsors should provide to allow biosimilar interchangeability, to asking whether there is any evidence suggesting that a biosimilar *cannot* be interchangeable. These three different views are all present in the results, exemplifying the lack of a common vision for substitution between reference and biosimilar products. In 2021, several EU regulators [38] reported their analysis of biosimilar European Public Assessment Reports and EMA post-marketing safety reports and concluded from their personal view that EU-approved biosimilars are interchangeable which is in line with our results. However, these regulators also describe that pharmacy-based substitution (see definition Table 1) of biosimilars is possible, which is contradicting some of the present results. One reason for the discrepancy could be the difference in time between the data collection and these regulators’ opinions [38], or alternatively that these regulators’ opinions do not illustrate all regulator opinions in the EU.

Another way of shedding light on the situation is to compare it to generics. The scientific question of likeness was also present when generic substitution was introduced [39]. This led to the emergence of pharmacokinetics to establish similarity for generics of small-molecule drugs, which is now considered basic science [39] and indeed also a key issue in biosimilar assessment. Based on the present results, it seems unlikely that regulators will ask for additional data or science to support biosimilar substitution. Thus, trust in the rigour of the existing EMA biosimilar approval system to support substitution is the only thing that remains to be communicated by regulators, although the publication by Kurki et al., 2021 [38] is a step on the way. Ultimately, the implementation of substitution depends on political incentives for member states as well as their health technology assessment (HTA) bodies’ reviews [40].

The pharmaceutical strategy from the European Commission (EC) [41] states that there are plans to review the pharmaceutical legislation, also regarding interchangeability of biosimilars, and that absence of automatic substitution can create market barriers that influence access to biosimilars. In addition, EMA’s regulatory science strategy [42] states that the EMA wishes to support trust and confidence in biosimilars among healthcare professionals and patients. Their means to aid trust and confidence is worded as “beyond data use” [42], which can be interpreted as not requiring additional data for biosimilars for this strategy. Although these two strategies show that the EC is working on the political and financial aspects of substitution, they also show that the EMA is not currently planned to play a larger role in the science underlying interchangeability beyond communicating the rigour of biosimilar approvals in the EU. We hope these efforts will include clarity on the scientific foundation for multiple switching of biosimilars as this is currently an essential missing piece. In light of the present results, the steps initiated by the EC and the EMA are needed to facilitate biosimilar substitution because biosimilar companies lack interest in substitution and are therefore unlikely to drive the change. Moreover, the development of interchangeability in the UK will be interesting to follow because a more “progressive” approach may be used, as previously seen for the UK biosimilar comparability trial requirements [24].

We urge EU and UK policymakers and regulators for clarity about their visions for biosimilar substitution. Some initiatives have been taken by the EC and EMA in their strategies, but more is needed if biosimilar substitution should become a reality. Positions and communications on biosimilars influence not only today’s biosimilar use, but also indicate their future role as envisioned by stakeholders. Their visions can gain momentum and become *“collectively held*, *institutionally stabilized*, *and publicly performed visions of desirable futures”* in line with Jasanoff’s theory of sociotechnical imaginaries [43]. Looking outside the EU, there are still varying approaches in different jurisdictions such as the switching study requirement by the US FDA and the naming policy prohibiting biosimilar substitution by the Japanese Pharmaceutical and Medical Devices Agency (PMDA) [44]. The new UK guidance from the MHRA also states that substitution is not possible. However, the Australian Pharmaceutical Benefits Advisory Committee flags individual biosimilar products as suitable for substitution [44]. We believe that obtaining a unified vision within the EU and in the UK could facilitate increased trust in biosimilars and give clarity on the future of biosimilars. Further, as the EU and the UK are frontrunners in the biosimilar field, their visions are likely to impact other regions.

Clinicians can expect the policy debate on biosimilar substitution to escalate in the near future. It is also likely that physicians’ attitudes on biosimilar substitution will be addressed. A recent systematic review [10] found that that 6–38% of physicians viewed biosimilars as interchangeable for the reference product, but another study found that 28% of rheumatologists believed that biosimilar and reference products cannot be interchanged at all [10, 13]. Further, such efforts may build on evidence that pharmacists have more education on medications compared with physicians and can therefore be educated further on substitutability [45].

Future research should investigate the existing knowledge of switching and substitution and narrow the knowledge gap regarding the effects of multiple switches between biologics [22]. Switching between biologics with the same active substance is already happening, potentially multiple times, in top-down driven systems such as in Norway and Denmark, where national bodies negotiate prices for biologics used in hospitals [46–48]. In these systems, automated transitions from reference products to biosimilars have led to substantial cost savings [4]. The subsequent contract renewal potentially introduces multiple switches for patients whenever another (biosimilar or reference) product has a lower price than the previously supplied product. Another system to study would be the substitution practice permitted in Australia. Studying these different systems would be a way of gaining knowledge of single and potentially multiple switches by capitalizing on the already existing real-world data.

A strength of the study is that it reflects views on interchangeability from different types of experts with various backgrounds and affiliations. This allowed capture of broad and deep data on the topic of interchangeability of biosimilars that is not possible with quantitative methods. It is possible that other expert views exist that were not captured, and thus saturation might not have been reached [49]. However, the results are data driven and documented by quotes. Four eligible interviewees were approached who subsequently declined to participate: three were prevented from participating due to in-house policy and one did not have the time. Telephone interviews have been criticized to affect the interview [50], however, we only observed more frequent clarification requests from interviewees and which were not obstructive of the flow or the responses during the interview. Further, the length of the interviews (median: 1h and 2 minutes) was not found to be short and the data were found to be rich and with depth based on the authors’ experiences with qualitative data. Thus, that the interviews were conducted via telephone did not influence the quality of the interviews. A main limitation is that this study was conducted prior to the COVID-19 pandemic and therefore opinions between now and then could have occurred [51]: this may be of interest to investigate in a future study. Another limitation is that we did not interview HTA bodies or physicians and that the interviews were conducted pre-Brexit and thus do not capture any possible differences in opinions between the UK and current EU regulators. Further, it is a limitation that it was not possible to recruit regulators from the FDA or EMA. Thus, there is a potential bias regarding the participating regulators’ opinions on the US system as they have only theoretical, rather than hands-on, experience with the system. An additional strength is that the authors have cross-disciplinary expertise that allows for a higher degree of reflexivity and nuanced interpretations of the complex field, building on the quality criteria by Malterud and Kitto et al. [52, 53]. The transferability of the findings is most likely restricted to the EU as other jurisdictions have other legal structures and frameworks for regulating interchangeability. However, given that the EU is a frontrunner in the biosimilar field, it is possible that other jurisdictions will find the study relevant as inspiration for debate of the issue.

## Conclusion

This study shows that the interviewed experts within EU national medicines agencies and the pharmaceutical industry view interchangeability as more than a scientific question of likeness between biosimilar and reference products. It also pertains to regulatory practices and trust. Participants were overall confident about biosimilar switching. However, their opinions differed about the scientific risk associated with substitution of biosimilars. For science to underpin substitution, almost all participants saw no need for additional data. Thus, it is unclear why biosimilar switching is viewed as scientifically safer than substitution. We urge European and UK policymakers and regulators to clarify the visions for biosimilar substitution and its potential to reduce the burden on strained healthcare systems. The positions of these two frontrunners are likely to influence other jurisdictions on the future of biosimilar use.

## Supporting information

S1 Table Interview guides. (DOCX)
