# Supplementary material for: Interchangeability of biosimilars: A study of expert views and visions regarding the science and substitution
Source: PLoS One. 2022 Jan 11;17(1):e0262537. doi: 10.1371/journal.pone.0262537 (PMC8751983; doi:10.1371/journal.pone.0262537)
Supplement: S1 Table — (DOCX) [file pone.0262537.s001.docx]

**S1 Table**

**Interview guides**

| **Interview guide – medicines authority regulators**  **Interview introduction**  Introduction to the interviewer and the project; the context of the study (PhD project); objective of the study; the topics of the interview. Information about anonymity and confidentiality.  Interviewer ask the interviewee to introduce their experience with biosimilars.  Interviewer ask for permission to tape-record and for written informed consent. Asking if any questions before the interview.  **INTERVIEW TOPIC 1. The incentives for the EU regulation of biosimilars:**   - What do you think are the 3 main incentives for the introduction of the regulation of biosimilars in the EU?   - Do you think one was more important than the others? - Wherefrom did the initiative for the legislation for introducing biosimilars come? - Do you think the current structure of this regulation fulfils these incentives? - Do you think the current structure of the regulation of biosimilars is adequate? - How do you think that the step-wise approach is functioning in practice?   **INTERVIEW TOPIC 2. Innovation**   - Do you think that the biosimilar regulation has influenced the incentive for innovation in new biologics? Why/Why not? - What is the incentive for companies to develop their first biosimilar? - What is the incentive for companies to develop their second or following biosimilar? - Do you consider biosimilars (developing new process and new product) to be re-producing the science made in the invention of the originator product or to be ‘new science’?   - Is it new knowledge if a company builds a new factory to make the same product?   - How do you see your organization to play a part in transmission of “know-how” knowledge?   **INTERVIEW TOPIC 3. Competition in the EU**   - How do you think that the competition in the biologics market is after introducing biosimilars?   - What would you like to change about it if you could?   - How do you think it could be changed? - Do you think that public health has benefitted from biosimilars? If so, why?     **INTERVIEW TOPIC 4. Comparability exercise vs. establishing biosimilarity**   - Do you see a scientific difference between the comparability exercise in relation to manufacturing changes compared to establishing biosimilarity for a proposed biosimilar? - Do you think that there are differences between requirements for establishing biosimilarity and approving manufacturing changes? - Do you think that the two types of procedures are handled similarly by medicines agencies?   - Is that handling adequate according to you?   **INTERVIEW TOPIC 5. Interchangeability of biosimilars**   - How do you see interchangeability of biosimilars in the EU? - How do you see interchangeability of biosimilars in the US? - Do you think that there is a scientific difference in interchangeability between EU and US? - How do you think that the interchangeability designation should be?   **INTERVIEW TOPIC 6. Comparison to the US**   - What do you think are the incentives of the biosimilar regulation in the US? - How do you think the current structure of this regulation fulfils this incentive?   **Interview outro**   - Do you see upcoming technological evolution/trends that would allow biosimilars to be identical to the originator biological? - Are there other consequences of the introduction of the biosimilar regulation that you find important and that we have not talked about? - What do you think is the most important that we have talked about? - Are there other relevant aspects that we have not talked about? - Are there any potential “landmines”/controversial aspects I need to be aware of?   Thank you so much for your participation this has been of great help! If I at a later point have more questions, can I reach out to you for an additional interview?  **Interview guide – pharmaceutical company participants**  **Interview introduction**  Introduction to the interviewer and the project; the context of the study (PhD project); objective of the study; the topics of the interview. Information about anonymity and confidentiality.  Interviewer ask the interviewee to introduce their experience with biosimilars  Interviewer ask for permission to tape-record and for written informed consent. Asking if any questions before the interview.   - If the interviewee is from a company, the following interview-start will be used: - Does company X currently market biosimilars? - Does company X have biosimilars in the pipeline or plan to have? - Does company X market so-called innovator-biologics? - Would you mostly say that company X is an ‘originator’ or a ‘biosimilar’ company?   - Does company X have other business units that are also producing biosimilars?   **INTERVIEW TOPIC 1. The incentives for the EU biosimilar regulation:**   - What do you think are the 3 main incentives for the introduction of the biosimilar regulation in EU?   - Do you think one was more important than the others? - Wherefrom did the initiative for the legislation for introducing biosimilars come? - Do you think the current structure of this regulation fulfils these incentives? - Do you think the current structure of the biosimilar regulation is adequate? - Companies with marketed biosimilars specific questions: - Have you undertaken a step-wise biosimilar development?   - Is this the general picture? - Did your view on the biosimilar approval pathway change after the decision on the biosimilar approval of *Product Z*, and if so how? - On basis of your experience with biosimilars, has company X had a change in incentive for continuing to develop biosimilars? If so, what caused this change? - Having the experience with successful biosimilar development and manufacturing, do you feel more or less incentivized to try developing a second? Why/Why not? - Companies only with marketed originator products specific questions: - Do the biosimilar regulation make company X consider developing biosimilars, why/why not?   **INTERVIEW TOPIC 2. Innovation**   - Do you think that the biosimilar regulation has influenced the incentive for innovation in new biologics? Why/Why not? - How is the incentive for companies to develop their first biosimilar? - How is the incentive for companies to develop their second or following biosimilar? - Do you consider biosimilars (developing new process and new product) to be re-producing the science made in the invention of the originator product or to be ‘new science’?   - Is it new knowledge if a company builds a new factory to make the same product?   - How do you see your organization to play a part in transmission of “know-how” knowledge? - Companies only with marketed originator products specific questions: - Have you considered to change your innovation focus from traditional biologic development to using new emerging technologies to avoid biosimilars being developed for your product?   **INTERVIEW TOPIC 3. Competition in the EU**   - How do you think that the competition in the biologics market is after introducing biosimilars?   - What would you like to change about it if you could?   - How do you think it could be changed? - Do you think that public health has benefitted from biosimilars? If so, why?     **INTERVIEW TOPIC 4. Comparability exercise vs. establishing biosimilarity**   - Do you see a scientific difference between the comparability exercise in relation to manufacturing changes compared to establishing biosimilarity for a proposed biosimilar? - Do you think that there is alignment between requirements for establishing biosimilarity and approving manufacturing changes? - Do you think that the two types of procedures are handled similarly by medicines agencies?   - Is that handling adequate according to you?   **INTERVIEW TOPIC 5. Interchangeability of biosimilars**   - How do you see interchangeability of biosimilars in the EU? - How do you see interchangeability of biosimilars in the US? - Do you think that there is a scientific difference in interchangeability between EU and US? - How do you think that the interchangeability designation should be?   **INTERVIEW TOPIC 6. Comparison to the US**   - What do you think are the incentives of the biosimilar regulation in the US? - How do you think the current structure of this regulation fulfils this incentive? - Companies with marketed biosimilars specific questions: - Did you experience differences in the step-wise approach for approval in the two juristictions? - Have you received scientific advice from both EMA and FDA during development, where these identical?   - If not, how did they differ?   **Interview outro**   - Do you see upcoming technological evolution/trends that would allow biosimilars to be identical to the originator biological? - Are there other consequences of the introduction of the biosimilar regulation that you find important and that we have not talked about? - What do you think is the most important that we have talked about? - Are there other relevant aspects that we have not talked about? - Are there any potential “landmines”/controversial aspects I need to be aware of?   Thank you so much for your participation this has been of great help! If I at a later point have more questions, can I reach out to you for an additional interview? |
| --- |
